# Intelligence

**Published:** 1807-07

**Authors:** 


### INTELLIGENCE.

A new method of curing those dreadful convulsions which carry off so many brave wounded soldiers, has been practised in the hospitals of Germany with great success. It was first resorted to by the late M. Stutz, a physician of eminence in Suabia, and he was led to this important discovery from the analogy of a simple fact. M. Humboldt had announced, in his Work upon the nerves, that on treating the nervous fibre alternately with opium and carbonate of potash, he made it pass five or six times from the highest degree of irritability to a state of perfect asthenia.

The

The method of M. Stutz, which has been employed with the great- success in the German hospitals, consisted in an alternate internal application of opium and carbonate of potash. It has been seen that when thirty-six grains of opium, administered in the space of twenty-four hours, produce no effect, the patient was considerably relieved by ten grains more of opium, employed after having given the alkaline solution. This new treatment of Tetanus is worthy of attention.

There is now living at Marseilles, a girl called Rosalia-Zaccharia Ferriol, aged ten years, and born at that city, of French parents, who possesses all the characters of the Albinos. The colour of her skin is of a dull white; her hair is straight and somewhat harsh to the touch, and is of a shining white colour, as are likewise her eye-lashes and eye-brows. Her eyes are large and rolling, the iris being of a clear blue with red streaks, and the cornea of a bright and vivid red. The sensibility of the visual organs is very great, the child not being able to bear much light, that of the sun obliging her to close her eyes. This girl, though much deformed in person, enjoys good health, and has never been afflicted with any disease except the small-pox. She is very fond of high seasoned food, is lively and intelligent; the father has chesnut-coloured hair, and appears to enjoy good health; the mother is a brunette, strong, and neither her nor her husband have ever been afflicted with any severe disorder; she has had five children, who are all living, but never during pregnancy was indisposed more than women usually are. All her children have, except the girl above described, chesnut coloured hair, and are perfectly well formed.

A traveller has presented to the Museum of Baltimore, brought by him from the banks of the Missouri, an enormous tooth of a Mammoth. He says, that, while engaged with other persons in researches relative to the existence of mines in the neighbourhood of the river, they found a space of about a quarter of a mile of extent wholly covered, to the depth of six feet, with bones of an enormous size. He offers to procure for any person who will pay him for the expence and trouble, a complete skeleton of the Mammoth, fifty-four feet in length, and twenty-two feet in height. Each of the jaw bones has eight enormous grinders.

The second volume of the Botanist's Guide through the Counties of Northumberland and Durham, will appear early in the present month. In this volume a considerable number of British lichens are now, for the first time, arranged, according to the Methodus Lichenum of Acharius; a copious Addenda to the first volume is prefixed; and to render the work more generally acceptable, an Index of English names is added. This volume completes the Flora of those Counties, and will contain about 1880 species.

Dr. Miller, Lecturer on Chemistry, at Edinburgh, has undertaken to prepare for the press, a new edition, in two volumes octavo, of Williams's Mineral Kingdom. He proposes carefully to to revise the original, to expunge all extraneous matter, to correct and polish the style, and to add the valuable discoveries that have been made in the Science of Mineralogy, since the publication of that Work. Dr. MILLER has made an actual survey of all the principal Mines of the kingdom, and may be supposed well qualified to execute this undertaking in a scientific manner.

Mr. WILLIAM TURNBULL, author of the Naval Surgeon, announces a system of British and French Surgery, medical and operative. Containing the most modern improvements in the science; arrranged on Clinical principles, and uniting anatomical information, so far as is necessary for the two subjects of Anatomy and Surgery to illustrate each other. The whole enriched with Plates, and original delineations, and to form three octavo volumes.

The following is a list of all the Cities in France which contain a population of thirty thousand people and upwards.

| | | | |
|---|---|---|---|
| Paris - - | 547,756 | Strasburgh - - | 49,056 |
| Marseilles - - | 96,413 | Cologne - - | 42,706 |
| Bourdeaux - - | 90,992 | Orleans - - | 41,937 |
| Lyons - - | 88,919 | Amicus - - | 41,279 |
| Rouen - - | 87,000 | Nismer - - | 39,594 |
| Turin - - | 79,000 | Bruges - - | 33,632 |
| Nantz - - | 77,162 | Angers - - | 33,000 |
| Brussels - - | 66,297 | Montpellier - - | 32,723 |
| Anvers - - | 56,318 | Metz - - | 32,099 |
| Ghent - - | 55,161 | Caen. - - | 30,923 |
| Lisle - - | 54,756 | Rheims - - | 50,225 |
| Toulouse - - | 50,171 | Alexandria - - | 30,000 |
| Liege - - | 50,000 | Clermont - - | 30,000 |

